# Care Management of Comorbid Medical and Psychiatric Illness: A Conceptual Framework for Improving Equity of Care

**DOI:** 10.1089/pop.2021.0366

**Published:** 2022-04-19

**Authors:** Lisa C. Rosenfeld, Philip Wang, Jackie Holland, Matthew Ruble, Taft Parsons, Hsiang Huang

**Affiliations:** ^1^ Department of Psychiatry, Cambridge Health Alliance, Cambridge, Massachusetts, USA.; ^2^ Department of Psychiatry, Harvard Medical School, Boston, Massachusetts, USA.; ^3^ Humana, Inc., Louisville, Kentucky, USA.

**Keywords:** behavioral health, care management, chronic disease, comorbid conditions, health disparities

## Abstract

Psychiatric and medical comorbidities are common among adults in the United States. Due to the complex interplay between medical and psychiatric illness, comorbidities result in substantial disparities in morbidity, mortality, and health care costs. There is, thus, both an ethical and fiscal imperative to develop care management programs to address the needs of individuals with comorbid conditions. Although there is substantial evidence supporting the use of care management for improving health outcomes for patients with chronic diseases, the majority of interventions described in the literature are condition-specific. Given the prevalence of comorbidities, the authors of this article reviewed the literature and drew on their clinical expertise to guide the development of future multimorbidity care management programs. Their review yielded one study of multimorbidity care management and two studies of multimorbidity collaborative care. The authors supplemented their findings by describing three key pillars of effective care management, as well as specific interventions to offer patients based on their psychiatric diagnoses and illness severity. The authors proposed short-, medium-, and long-term indicators to measure and track the impact of care management programs on disparities in care. Future studies are needed to identify which elements of existing multimorbidity collaborative care models are active ingredients, as well as which of the suggested supplemental interventions offer the greatest value.

## Background

Psychiatric and medical comorbidities are extremely common among adults in the United States, with 68% of those with mental disorders also having medical conditions and 29% of those with medical conditions also having mental disorders.^1^ Due to the complex interplay between medical and psychiatric illness, comorbidities result in substantial health disparities.

People with comorbid conditions tend to have more severe symptoms and greater functional impairment than the general population.^1^ Further, these patients have a 2- to 4-fold elevated risk of premature mortality, typically due to cardiovascular disease, and greater health care costs.^2–4^ Bearing these facts in mind, there is an ethical and fiscal imperative to develop interventions that address the needs of individuals with comorbid medical and psychiatric conditions (hereafter referred to as “comorbidities”), to reduce both health disparities and care costs.

One evidence-based approach to improving the quality of care and health outcomes for people with chronic diseases is care management. Care managers support patients through assessment, care planning, and care coordination.^5^ One specific model of care that uses care managers and has shown promise in addressing the needs of patients with psychiatric illness is collaborative care. This model is a team-based approach that emphasizes using care managers to empower patients to manage their own illnesses; provide decision support tools to ensure measurement-guided, evidence-based care; coordinate the flow of information between various members of a patient's care team; and employ population health strategies, such as patient registries, to track outcomes over time.^6^ These interventions are guided by case reviews between the care manager and a consulting mental health specialist (typically a psychiatrist).

Although there is substantial evidence supporting the effectiveness of models of care that utilize care managers, including for reducing disparities among racial and ethnic minorities,^7^ the majority of interventions described in the literature are disease-specific. Given the prevalence of comorbidities in the general population, single-disease care management programs may be less practical to implement and less efficient for improving outcomes than programs that target multiple conditions at once.

For this reason, the authors of this article reviewed the literature on care management models that target both medical and psychiatric outcomes with the goal of enhancing an existing insurance-based care management program for people with comorbidities. The findings of this review are summarized in the next section. Given the relative paucity of literature on interventions that measure both medical and psychiatric outcomes, the authors supplemented their review by outlining a conceptual framework for the development of care management programs serving individuals with mental and physical comorbidities.

Although the framework described below was initially created to serve the needs of Medicare-eligible beneficiaries, the authors believe these recommendations are broadly applicable and important considerations when developing any care management program that aims at improving health equity for individuals with medical and psychiatric comorbidities.

## Evidence-Based Models

The authors reviewed the literature to identify studies of care management programs that aimed at improving medical and psychiatric outcomes for patients with comorbidities. Although there were numerous studies examining the impact of such programs on mental health outcomes^6^ or non-specific scores of physical well-being or functional status,^8,9^ there were relatively few that measured outcomes for both medical and psychiatric conditions.

Of the studies that did measure both sets of outcomes, the interventions fell predominantly into one of two categories: traditional care management programs and collaborative care interventions.

With regard to traditional care management, the authors identified one randomized controlled trial^10^ that followed two pilot studies,^11,12^ all of which were rated as “fair” in quality in a systematic review of care management interventions targeting multimorbidity.^13^ This randomized controlled trial examined whether integrating depression and diabetes care management improved adherence to medication regimens, as well as outcomes for both depression and diabetes.

The trial included 180 participants who were treated at a single community-based primary care practice in West Philadelphia. The intervention utilized two research coordinators (one bachelors- and one masters-level) as care managers. The care managers received training on pharmacotherapy for depression and diabetes, as well as on culturally competent evidence-based strategies for quickly building rapport.

The role of the care managers was to help patients recognize depression in the context of their medical illness, explain the rationale for treatment with medications, monitor medication adherence, and help address medication side effects. In addition, medication adherence was tracked by using electronic devices that registered when pill bottles were opened. Over the 3-month study period, the care managers met with study participants three times in-person for 30 minutes, and two times by phone for 15 minutes. Baseline demographic and clinical features did not differ between the two study arms.

At the 3-month follow-up, the trial found statistically significant improvements in medication adherence, depression scores, and diabetes outcomes for participants in the intervention group as compared with the usual care group. Although the limitations of this study include questions about generalizability, restriction to patients with comorbid depression and diabetes, and a short follow-up window, strengths include its low level of resource intensiveness (given no involvement from a mental health specialist) and its inclusion of majority African American study participants, who have long suffered from health disparities.^14^

With regard to the collaborative care model, the first of two studies was TEAMcare,^15^ which was rated as “good” quality in the aforementioned systematic review of multimorbidity care management programs. The TEAMcare study was an offshoot of the Pathways study (a program aimed at improving depression and diabetes outcomes through enhanced depression care^16^). The creators of Pathways hypothesized that un- or under-treated depression interfered with self-care behaviors, and that reductions in depressive symptoms would lead to improvements in diet, exercise, and medication adherence, which, in turn, would result in improvements in diabetes outcomes.

However, the Pathways study ultimately found that depression care management alone did not result in improved diabetes outcomes. As a result, TEAMcare was developed to test whether a primary-care based care management program that simultaneously targeted depression and common comorbidities—specifically, diabetes, hypertension, and hyperlipidemia—could improve outcomes across these diagnoses.

TEAMcare enrolled 214 patients (mean age 57) from 14 primary care clinics in Western Washington. The intervention used nurse care managers who received a 2-day course in depression care, with emphasis on diagnosis, treatment, and specific therapeutic interventions, including motivational interviewing and behavioral activation. They also received a refresher course in active management of the three medical conditions listed earlier.

The nurse care managers led structured visits with patients every 2 to 4 weeks, focusing on disease self-management, development of individualized care plans, careful monitoring of outcome measures (such as hemoglobin A1c for diabetes), and relentless adjustment of interventions to achieve the desired health outcomes, also known as “treat-to-target.” The care managers also maintained a patient registry that was reviewed in weekly supervision with a psychiatrist, primary care physician, and psychologist, all with an eye to patients who were new or not responding to treatment as expected.

Over the course of the 12-month study, intervention group participants experienced greater improvements in the composite primary outcome measure—which included SCL-20, hemoglobin A1c, low-density lipoprotein, and systolic blood pressure—than control group participants.^15^ Intervention group participants also experienced greater improvements in secondary measures, such as quality of life and satisfaction with care. Further, in a 2-year follow-up study assessing cost-effectiveness, the intervention group had a nearly $600 reduction in outpatient costs, even after accounting for the costs of the intervention.^17^

Thus, TEAMcare demonstrated the cost-effectiveness of a primary care-based program in which one medically trained nurse care manager had regular access to a consulting mental health specialist and simultaneously addressed patients' psychiatric and medical gaps in care.

The second collaborative care study, COMPASS, was a dissemination project that included a much larger sample size (*n* = 3609) across a broader range of settings.^18,19^ COMPASS included 18 medical groups spanning 172 primary care and multi-specialty clinics, with between 51 and 684 participants each, across 8 states (in a mix of rural, suburban, and urban settings).

Although the intervention offered in COMPASS was modeled directly after TEAMcare, it did allow for some customization to suit local needs and constraints. For instance, treat-to-target protocols were adapted to regional prescribing practices and care managers were recruited from a variety of backgrounds and specialties. Some offered blended in-person and phone-based care management, whereas others were exclusively phone-based.

Finally, some opted to add members to their systematic case review teams, such as pharmacists, social workers, and diabetes educators. As with TEAMcare, COMPASS was associated with clinically and statistically significant improvements in both psychiatric and medical outcomes. Outcomes did not vary based on the training or background of the care managers.

Given the relative paucity of data, combined with the great need to serve patients with comorbid medical and psychiatric conditions, the authors of this article supplemented the earlier cited review with a conceptual framework that includes “General considerations” and “Specific interventions” to keep in mind when developing care management programs for this patient population.

## General Considerations

The considerations that follow are informed by the current literature review and by clinical expertise among the authors. The first consideration is whether to match patients with one or two care managers to address their medical and psychiatric disorders. The authors strongly recommend pairing each patient with a single care manager. Doing so will reduce the risk of miscommunication and duplication of services, thereby leading to more efficient care.

It will also foster a sense among patients that their assigned care manager is their “go-to” person, which will likely breed trust and cut down on confusion. Finally, and perhaps most importantly, assigning a single care manager will allow programs to be nimble enough to address patients' care gaps in the order that is most important to them, which is pivotal to upholding the principle of patient autonomy.

**FIG. 1.**
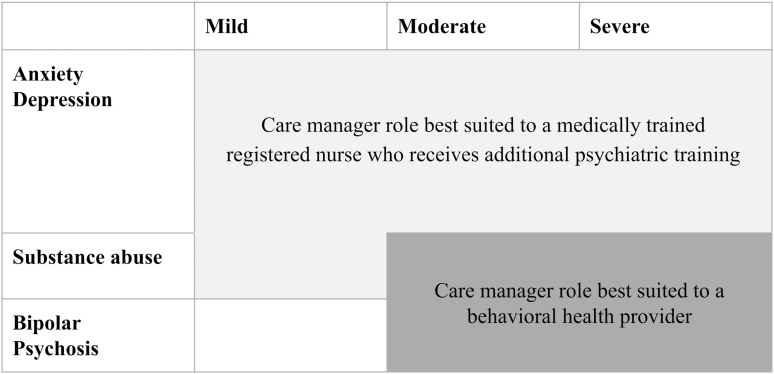
Recommended qualifications for care manager, stratified by patient's primary psychiatric disorder.

The second consideration is what qualifications a care manager must have to serve in this role (Fig. 1). All else being equal, this should be determined by a patient's primary psychiatric diagnosis. For individuals with mild to moderate anxiety, depression, and substance use disorders (SUDs), registered nurses with competence in assessing clinical status and medication adherence and tolerability would ideally serve in the role of care manager.

Alternatively, for patients with serious and persistent mental illness (such as bipolar or schizophrenia) or severe mood, anxiety, or SUDs, care managers with behavioral health expertise (such as a social worker or licensed mental health counselor) would be better suited for the role.^6^ Even more important than the specialization of care managers,^20^ however, is ensuring (1) access to adequate training and continuing education, (2) an even distribution of complex cases across care managers to reduce burnout and foster a sense of efficacy, and (3) consistent supervision that includes case reviews with a mental health specialist,^21^ primary care provider, and clinical pharmacist.^22,23^

Of note, the provision of regular supervision with specialists has previously been identified as an active ingredient in the collaborative care model.^24,25^ Ideally, this supervision should focus on planning interventions for new patients, problem solving for patients not responding to interventions as expected, gathering input on medication adjustments and management of side effects, and getting guidance on how to approach challenging patient encounters.

If possible, programs should also offer as-needed supervision of care managers with medical specialists to consult on patients with rare or complex conditions. This additional supervision could be conducted in groups to facilitate ongoing education, mutual support, and efficient use of specialist time.

The third consideration in the design of a care management program is how to prioritize among gaps in care for individuals with disparities across multiple health conditions. In general, the authors recommend following a patient's lead, as doing so will enhance buy-in to a process that will likely require behavior change and may even lead to a virtuous cycle in which making progress on one goal leads to increased confidence in one's ability to make progress on another.

Further, honoring individual preferences about which gaps to tackle first will allow care managers to focus on behavioral medicine principles that cut across diseases, such as disease self-monitoring,^26^ medication adherence,^10,27^ healthy diet,^28^ and increased physical activity,^27,28^ rather than on the specifics of any one condition. In rare cases where members do not have a preference or are unable to communicate a clear preference, behavioral health needs should be addressed before physical needs in patients with severe pathology, such as bipolar affective disorder and schizophrenia.

For all other patients, the authors agree with the approach taken in the studies identified in the literature review, which was to simultaneously address patients' behavioral health needs and common medical risk factors for downstream events, namely hypertension, hyperlipidemia, diabetes, and tobacco use.

**FIG. 2.**
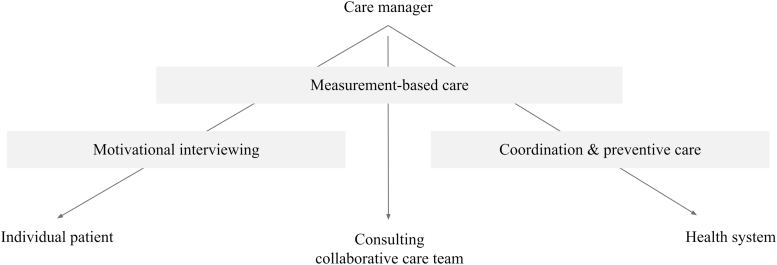
Three key pillars of effective care management.

The final consideration is what to include in the training of care managers. The authors argue that training is critical and should focus on three key pillars of effective care management (Fig. 2), which are discussed next in detail:

1. Measurement-based care
2. Motivational interviewing
3. Care coordination

Measurement-based care (MBC) is “the systematic administration of symptom rating scales and use of the results to drive clinical decision making.” Although MBC has been more widely adopted in medical than psychiatric models of care, there is good evidence to support its use in mental health care. A systematic review of 51 studies provided ample evidence that MBC, when conducted frequently and synced with care, led to improved mental health outcomes as compared with usual care.^29^

The MBC can be used to systematically identify patients for interventions, which has been identified as an active ingredient of collaborative care models in multiple studies.^20,25^ Further, MBC can guide conversations (1) with patients about how treatment is progressing and to motivate change, (2) when reviewing caseloads with the entire care management team, especially to help in identifying people not responding to treatment as expected, and (3) to frame treatment recommendations that care managers make to patients' individual physicians and other care providers.

Finally, although the purpose of MBC is to guide the treatment of individual patients, an added benefit is that the data collected can also be used to assess the impact of care management programs more broadly, which is discussed in more detail under the Measuring Impact section.

Motivational interviewing (MI) is a therapeutic technique that aims at resolving ambivalence by tapping into a person's individual reasons for behavior change.^30^ Although MI was originally developed for the treatment of SUDs, there is now robust literature supporting its use in a range of applications, such as weight loss, blood pressure control, cholesterol control, and treatment adherence. A meta-analysis of MI revealed that encounters as brief as 15 minutes can lead to significant outcomes, and that more encounters are associated with a greater impact.^30^ This same analysis revealed that outcomes did not depend on the level of training of the person performing MI. By implementing MI, care managers can help patients identify personalized treatment goals, improve treatment engagement, and make healthy lifestyle choices.

Finally, poor communication between medical and mental health care systems results in health disparities for individuals with comorbid conditions.^31^ Thus, coordinating care by ensuring the timely flow of information between medical and mental health providers regarding upcoming appointment times, recent visits, and medication changes is highly valuable. To bring care coordination to the next level, care managers should focus on prevention.

At its most basic, this means ensuring access to preventive services such as immunizations and cancer screenings, which people with comorbidities have historically received at lower rates than the general population.^32,33^ Further, this involves monitoring for downstream complications of psychiatric medications, such as metabolic syndrome, which result in morbidity, mortality, and high cost care.^34^ Finally, care teams should work to prevent polypharmacy, which results in not only decreased medication adherence but also drug–drug interactions that lead to medication toxicity, delirium, falls, urinary incontinence, and avoidable admissions.^35^

## Specific Interventions

**FIG. 3.**
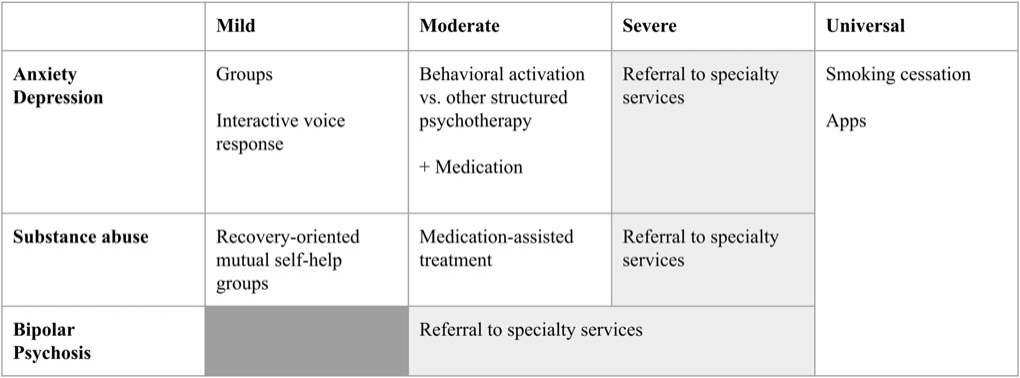
Recommended evidence-based interventions, stratified by psychiatric diagnosis and illness severity.

In this section, the authors recommend specific interventions that have the potential to improve outcomes and reduce disparities if incorporated into care management programs for people with medical and psychiatric comorbidities. Some of these interventions have universal applicability, whereas others should be tailored to an individual's psychiatric diagnosis and illness severity (Fig. 3). The list given next, which includes evidence-based, high value interventions, is not exhaustive. Further, the boundaries delineated between the sub-populations of patients listed are not rigid.

### Universal interventions

The authors strongly recommend that any care management program serving individuals with comorbidities include an evidence-based cigarette smoking cessation intervention. Tobacco use is the leading preventable cause of death and a risk factor for some of the most common conditions among individuals with medical and psychiatric comorbidities.^36^ Smoking is more prevalent among people with psychiatric disorders than for people in the general population, thereby contributing to health disparities.^37^ The majority of cigarette smokers want to quit, but they need support to do so.^38^

Effective smoking cessation treatments exist, and people who succeed in quitting experience improvements in their mood and anxiety, regardless of whether they have an underlying psychiatric illness.^39,40^ Finally, smoking cessation programs are an excellent return on investment. Studies estimate that they cost just $0.50 per person-month to operate,^41^ result in $9800 lifetime savings,^42^ and can pay for themselves in as little as 3 years.^43^

All eligible patients should also be offered a curated list of health apps. Two systematic reviews of health apps for anxiety and depression, respectively, show early evidence of efficacy in alleviating symptoms.^44,45^ Although the available data are less compelling for apps to help with weight management and substance use, the authors of this article have worked with patients who have responded to these sorts of apps. Given the low-cost, if not free, nature of health apps, there is potential benefit and little risk in making a variety of health apps broadly available.

### Interventions for mild anxiety and depression

Many cases of mild anxiety and depression are the product of social isolation,^46^ a problem that has become increasingly prevalent in the context of the COVID-19 pandemic.^47^ Certain groups—such as immigrants, elderly individuals, and victims of abuse—are at a particularly high risk of loneliness, resulting in mood and anxiety problems.^46^ Prior studies have observed that social deprivation can pose as great a threat to both mental and physical health as obesity and smoking.^48^

For this reason, the authors believe that offering interventions that increase social connectedness to patients whose primary behavioral health need is mild anxiety or depression is a worthwhile investment. One such intervention is group therapy, which is cost-effective, has evidence across a range of psychiatric disorders, and may offer particular benefit for patients who are homebound, such as elderly individuals with physical disabilities.^49^

Another possible intervention is interactive voice response (IVR) technology, a robocaller that can adapt its messages based on patient feedback and can offer supportive messages, track and encourage adherence to treatment, and triage to human support if needed or requested.^50^ Despite being minimally resource intensive, IVR technology is sufficiently lifelike that patients report feeling cared for and cheered up by IVR calls, with corresponding improvements in depression measures.^51^

### Interventions for moderate anxiety and depression

For patients with moderate anxiety and depression, a combination of brief therapeutic interventions carried out by the care manager, referral to structured psychotherapy with a specialist, and medication management should be offered based on patient preference.

Regarding therapeutic modalities, care managers could offer behavioral activation, an evidence-based intervention that discourages avoidance behaviors and encourages engagement in pleasurable activities. Studies of behavioral activation reveal evidence of equal efficacy when compared with cognitive behavioral therapy (CBT), typically considered the gold standard therapeutic intervention for anxiety and depression, even when administered by junior mental health workers.^52^

Alternatively, if organizations are unable to train care managers in behavioral activation, care managers could instead assist with referrals to structured psychotherapies, which have been identified as an active ingredient in the collaborative care model.^20^ Evidence-based therapies include cognitive interventions (such as CBT or mindfulness-based stress reduction), skills-based interventions (such as problem-solving therapy or pain management), and exposure therapy.^53,54^

Regarding medications for anxiety or depression, care management programs could either use an existing algorithm—such as the one in TEAMcare, Harvard South Shore's Psychopharmacology Algorithm Project,^55^ or the Texas Medication Algorithm Project^56^—or develop treatment protocols that reflect local prescribing practices. Regardless of the medications selected, the algorithms should utilize the treat-to-target approach previously described, with the goal of finding the lowest effective dose to achieve symptom remission.

### Interventions for mild SUDs

For patients with mild SUDs, care managers can provide education about recovery-oriented mutual self-help groups, such as Alcoholics Anonymous (AA), Narcotics Anonymous (NA), and Self-Management and Recovery Training (SMART) Recovery. A Cochrane Review of 27 studies with more than 10,000 participants concluded that AA and other similar programs were at least as effective, if not more effective, than other established treatments for SUDs, such as CBT.^57^ This review further concluded that these programs produced substantial health care cost savings among people with alcohol use disorder. With more than 60,000 meetings of varying shapes and sizes in the United States, care managers should focus on helping patients identify meetings that are a good fit and problem-solving barriers to attendance.

### Interventions for moderate SUDs

Patients with moderate SUDs should be offered the same education about mutual self-help groups as described earlier and should also be offered referral to medication-assisted treatment (MAT). MAT is typically available through specialty addiction or pain clinics, or through primary care offices with specially licensed providers (sometimes referred to as office-based addiction treatment, or OBAT). MAT should be offered to this group not only because there is clear evidence that it saves lives, but also because MAT has been shown to decrease the need for detoxes and treatment of medical sequelae of SUDs, such as HIV or hepatitis, all of which add to rising health care costs.^58,59^

### Interventions for severe psychiatric conditions

For patients with severe pathology—including bipolar disorder, psychosis, and severe depression, anxiety, or SUDs—care managers should prioritize referral to specialty care in psychiatry and then focus their attention on engagement in care, medication adherence, and careful monitoring for medication-related toxicity.

## Measuring Impact

**FIG. 4.**
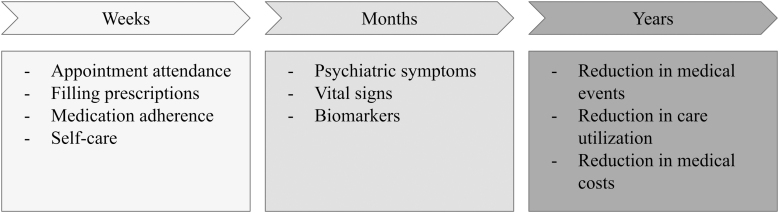
Indicators to measure the impact of care management programs.

To determine whether the aforementioned interventions are having the desired effect on gaps in care for individuals with medical and psychiatric comorbidities, the authors recommend tracking the following short-, medium-, and long-term indicators (Fig. 4).

On the order of weeks, one might observe changes in no-shows, prescription fill rates, medication adherence, and other health behaviors such as diet and exercise (“short-term indicators”). These changes could be measured by using a combination of data from billing, pharmacies, self-report, and laboratories. Insurers or health systems might also consider covering the cost of electronic pill boxes and wearables such as pedometers, which—with patient consent—may be able to transmit data directly to care providers or medical records.

On the order of months, one might observe changes in psychiatric symptoms, vital signs, and biomarkers of medical illness (“medium-term indicators”).^15^ These changes could be tracked through a combination of patient-reported outcome measures, vital sign measurement, and laboratory data. Insurers or health systems might consider covering the cost of durable medical equipment, such as glucose and blood pressure monitors, which can aid patients in disease self-management and may also be able to transmit data to care providers or systems.

Some of the most important outcomes—such as reductions in medical events, care needs, and health care spending—could take one or more years to observe a difference (“long-term indicators”). For instance, the TEAMcare model demonstrated reductions in outpatient care utilization and spending over the course of two years.^17^ Similarly, a study of collaborative care for bipolar disorder revealed reductions in care utilization after two years and reductions in medical spending emerged over the course of three .^60^

Finally, one study comparing usual care with a team-based care model in which care managers assisted with patient outreach and care coordination found reduced rates of primary care visits, ambulatory care sensitive visits, emergency department visits, and hospital admissions among patients in the intervention arm over the 3-year study period.^61^

Although health care systems with limited resources at their disposal might be reluctant to invest in comprehensive care management programs that could take years to demonstrate impact and cost savings, it is worth noting that medium-term indicators such as those listed earlier can provide insight into whether downstream impact is likely to occur.

For instance, in a UK study of a 1-year diabetes intervention with more than 4000 participants, post-trial monitoring revealed sustained risk reductions in diabetes-related end point, microvascular disease, myocardial infarction, and all-cause mortality 10 years after the study ended, even though between-group differences in hemoglobin A1c were lost after the first year.^62^

With this finding in mind, it stands to reason that if care management interventions can lead to improvements in vital signs, biomarkers, treatment engagement, and health behaviors that persist for more than a year, the resulting health improvements and reductions in care costs would be even greater.

## Moving Forward

In conclusion, many adults in the United States have comorbid medical and psychiatric conditions, and these individuals suffer from disparities in morbidity, mortality, and social and occupational functioning. Care management programs have the potential to address gaps in care and improve outcomes for individuals with comorbidities. Despite ample evidence for disease-specific care management programs, the evidence for multimorbidity care management is limited. The review described in this article yielded one study of traditional care management and two of collaborative care programs that simultaneously targeted medical and psychiatric outcomes.

Given the relative dearth of literature on multimorbidity care management, the authors supplemented their review by outlining the pillars of effective care management, which include measurement-based care, motivational interviewing, and care coordination. They also made recommendations regarding specific interventions that could be offered based on an individual's psychiatric diagnosis and illness severity.

As the recommendations outlined here were originally intended to enhance an existing insurance-based care management program, the suggested interventions were limited to what could reasonably be offered by care managers who might not be directly embedded in a patient's primary care team.

Future studies should focus on how to effectively implement and disseminate the core features of existing multimorbidity care management models. They should also build upon prior mental health-focused studies examining which components of the interventions cited earlier offer the greatest value, in what contexts, and for which sub-populations.^20,25^ Finally, they should evaluate the relative impact of care management programs for individuals with comorbid medical and psychiatric conditions as compared with the direct provision of mental health services in the primary care setting (ie, integrated care). Only once these questions are answered will we truly be able to make progress on providing equitable, high-quality care to individuals with medical and psychiatric comorbidities.
